# Supplementary material for: Dynalign II: common secondary structure prediction for RNA homologs with domain insertions
Source: Nucleic Acids Res. 2014 Nov 21;42(22):13939–48. doi: 10.1093/nar/gku1172 (PMC4267632; doi:10.1093/nar/gku1172)
Supplement: SUPPLEMENTARY DATA [file supp_gku1172_nar-02021-z-2014-File012.zip › manual/RNA_class/html/_dynalign__class_8cpp.html]

RNAstructure Classes: F:/RNAstructure-co-from-repository/RNAstructure/RNA\_class/Dynalign\_class.cpp File Reference

- Main Page
- Classes
- Files

- File List
- File Members

# F:/RNAstructure-co-from-repository/RNAstructure/RNA\_class/Dynalign\_class.cpp File Reference

`#include <iostream>`  
`#include "Dynalign_object.h"`  

|  |  |
| --- | --- |
|  |  |
| Functions | |
| int | main (int argc, char \*argv[]) |

---

## Function Documentation

|  |  |  |  |  |
| --- | --- | --- | --- | --- |
| int main | ( | int | *argc*, |  |
|  |  | char \* | *argv*[] |  |
|  | ) |  |  |  |

---

Generated on Tue Jul 30 10:52:29 2013 for RNAstructure Classes by 
 1.5.7.1
